# Isoflurane Potentiation of GABA_A_ Receptors Is Reduced but Not Eliminated by the β3(N265M) Mutation

**DOI:** 10.3390/ijms21249534

**Published:** 2020-12-15

**Authors:** Chong Lor, Misha Perouansky, Robert A. Pearce

**Affiliations:** Department of Anesthesiology, University of Wisconsin, Madison, WI 53705, USA; clor2@wisc.edu (C.L.); mperouansky@wisc.edu (M.P.)

**Keywords:** anesthetic action, general anesthesia, unconsciousness, amnesia, GABA, intravenous anesthetics, volatile anesthetics

## Abstract

**Background:** Mice carrying the GABA_A_ receptor β3(N265M) point mutation, which renders receptors incorporating β3-subunits insensitive to many general anesthetics, have been used experimentally to link modulation of different receptor subtypes to distinct behavioral endpoints. Remarkably, however, the effect of the mutation on the susceptibility to modulation by isoflurane (a standard reference agent for inhalational vapors) has never been tested directly. Therefore, we compared the modulation by isoflurane of expressed α5β3(N265M)γ2L receptors with their wild type counterparts. **Methods:** Using whole-cell electrophysiological recording and rapid solution exchange techniques, we tested the effects of isoflurane at concentrations ranging from 80 μM to 320 μM on currents activated by 1 μM GABA. We measured drug modulation of wild-type α5β3γ2L GABA_A_ receptors and their counterparts harboring the β3(N265M) mutation. **Results:** Currents elicited by GABA were enhanced two- to four-fold by isoflurane, in a concentration-dependent manner. Under the same conditions, receptors incorporating the β3(N265M) mutation were enhanced by approximately 1.5- to two-fold; i.e., modulation by isoflurane was attenuated by approximately one-half. Direct activation by isoflurane was also present in mutant receptors but also attenuated. **Conclusions:** In contrast to the complete insensitivity of β3(N265M) mutant receptors to etomidate and propofol, the mutation has only a partial effect on receptor modulation by isoflurane. Therefore, the persistence of isoflurane effects in mutant mice does not exclude a possible contribution of β3-GABA_A_ receptors.

## 1. Introduction

The ionotropic γ-aminobutyric acid receptor (GABA_A_R) remains a target of considerable interest in understanding the mechanism of action of general anesthetics [1,2,3]. To link modulation of specific subtypes of GABA_A_Rs to the various endpoints of anesthesia, a fruitful experimental strategy has utilized mice carrying point mutations of individual receptor subunits that alter or eliminate anesthetic sensitivity; by comparing drug effects in wild-type versus mutant animals, the role of the specific receptor subtypes can thereby be tested. For the injectable GABAergic anesthetics etomidate and propofol, this approach has linked GABA_A_Rs that incorporate β2 subunits to sedation and β3 subunits to loss of righting reflex, respiratory depression, and suppression of the hindlimb withdrawal reflex (“immobilization”) [4,5,6].

A similar strategy has been followed for the potent inhalational agents, including isoflurane. Mice carrying α1- or α2-subunit mutations weakly resisted isoflurane’s suppression of the righting reflex, but they did not differ from wild-type mice in tests of isoflurane-induced immobilization or memory [7,8]. Similarly, mice carrying the β3(N265M) mutation partially resisted isoflurane-induced immobilization [9,10], but memory suppression did not differ from wild-type mice [10]. Taken together, these findings have been taken to indicate that GABA_A_R modulation contributes modestly to the ability of isoflurane to suppress movement but not to its ability to block memory.

One important caveat to this last conclusion is that in vitro studies of β3(N265M) receptor modulation by isoflurane are lacking. In other studies of recombinant receptors, the β3(N265M) mutation strongly attenuated modulation of α2β3γ2 receptors by enflurane [11], and mutating the analogous residue of the β1 subunit completely prevented isoflurane from modulating α2β1(S265I) receptors [12]. However, many factors can influence pharmacological characteristics of mutated receptors, including the amino acid used to replace the native residue, the choice of the other subunits with which the mutant subunit is expressed, and the specific inhaled agent that is applied [11,12,13]. Given the critical nature of the assumption that the β3(N265M) mutation also eliminates sensitivity to isoflurane, the lack of a direct test of the effect of the mutation on isoflurane modulation constitutes an important knowledge gap.

To fill this gap, we tested the sensitivity of recombinant β3(N265M)-containing receptors to isoflurane. We chose to co-express β3 subunits with α5 and γ2L subunits because this combination is preferentially expressed in the hippocampus [14,15] and it is a plausible target for anesthetic interference with memory formation [16,17]. We found that the mutation reduced, but did not eliminate, isoflurane modulation. These results suggest that the conclusion that β3-GABA_A_Rs do not contribute to isoflurane-induced amnesia, and only contribute modestly to immobility, may be unwarranted.

## 2. Results

### 2.1. Isoflurane Modulation of GABA—Elicited Currents

**Figure 1:**
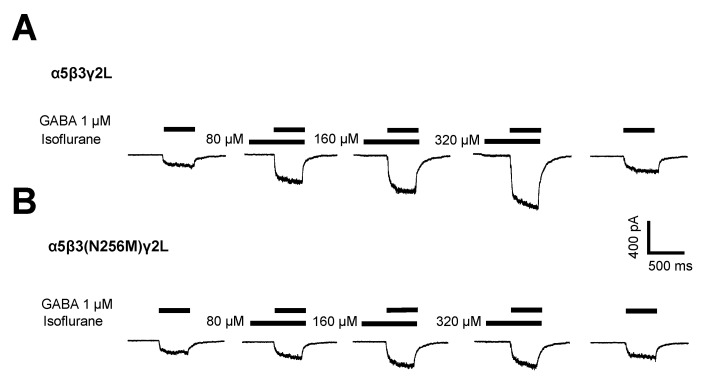
Activation and modulation of GABA-elicited currents by isoflurane in wild-type and mutant GABA_A_Rs. Traces show whole-cell currents elicited by 500 ms applications of isoflurane alone followed by isoflurane plus 1 μM GABA, for (**A**) α5β3γ2L and (**B**) α5β3(N265M)γ2L recombinant receptors expressed in HEK293 cells.

**Figure 2:**
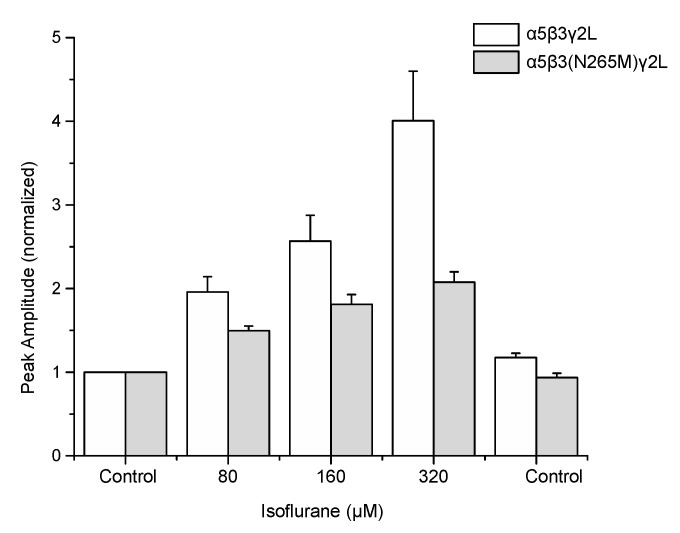
The β3(N265M) mutation attenuates but does not eliminate isoflurane modulation of α5β3γ2L receptors. Peak current amplitude elicited by 1 μM GABA in the presence of isoflurane at concentrations ranging from 80 μM (approximately EC50 for amnesia) to 320 μM (approximately EC50 for immobility) were normalized to control (isoflurane-free) responses. Isoflurane significantly enhanced GABA-elicited responses at all concentrations tested for both genotypes (*p* < 0.001 for all groups, z-test vs. a value of “1”, *n* = 5–6 for all groups), and enhancement of responses was significantly greater for wild-type than mutant receptors at all isoflurane concentrations tested (80 μM, *p* = 0.031; 160 μM, *p* = 0.034; 320 μM, *p* = 0.015; unpaired one-sided Students’ *t*-test, *n* = 5–6 for all groups).

Application of 1 μM GABA to recombinant GABA_A_Rs induced currents through both wild-type (α5β3γ2L) and mutant (α5β3(N265M)γ2L) receptors (Figure 1A). Peak currents ranged from −61 to −1205 pA and from −65 to −693 pA for wild-type and mutant receptors, respectively, reflecting the lesser potency of GABA at mutant receptors. [18] Co-application of isoflurane resulted in a dose-dependent, reversible potentiation of the currents in both wild-type and β3(N265M) receptors (Figure 1B). At the highest concentration of isoflurane tested (320 μM), responses to GABA ranged from −263 to −3612 for wild-type and −129 to −1491 pA for β3(N265M) mutant receptors. A summary of the modulatory effect of isoflurane on GABA-elicited currents in wild-type and mutant receptors is shown in Figure 2.

### 2.2. Direct Activation of GABA_A_ Receptors by Isoflurane

**Figure 3:**
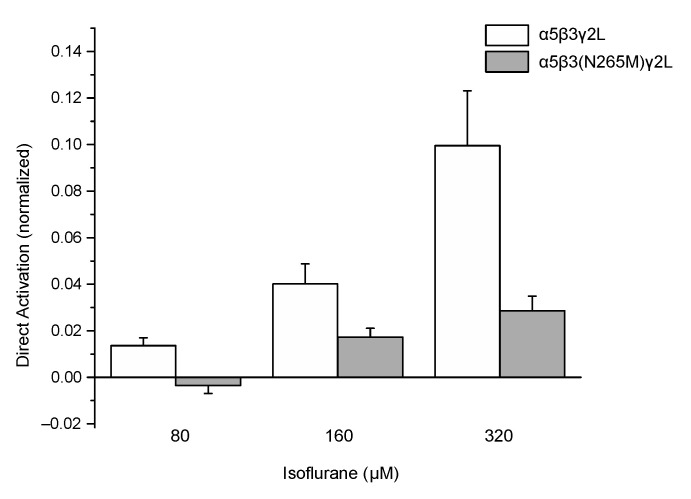
The β3(N265M) mutation attenuates but does not eliminate direct activation of α5β3γ2L receptors by isoflurane. Peak current amplitude elicited by isoflurane at concentrations ranging from 80 μM to 320 μM, in the absence of GABA, were normalized to peak responses to control (isoflurane-free) responses to 1 μM GABA. Isoflurane produced small but significant levels of current at all concentrations tested for wild-type receptors, and at the two higher concentrations for β3-N265M receptors (*p* < 0.001, z-test vs. a value of “0”, *n* = 5 for all groups), but not at 80 μM isoflurane for β3-N265M receptors (*p* = 0.81, z-test vs. a value of “0”, *n* = 5). Differences between genotypes were statistically significant at all concentrations tested (80 μM, *p* = 0.019; 160 μM, *p* = 0.031; 320 μM, *p* = 0.018; Students’ *t*-test, *n* = 5 for all groups).

Application of isoflurane alone (i.e., in the absence of GABA) weakly activated wild-type receptors (Figure 3, open bars), as reported previously [19]. This effect was attenuated in mutant receptors compared to wild-type (Figure 3, filled bars). At the highest concentration tested (320 μM), current amplitudes ranged from −11 to −159 pA for cells expressing wild-type receptors and from 0 to −15 pA at −60 mV for cells expressing mutant receptors. A summary of the direct activation by isoflurane of currents from wild-type and mutant receptors is shown in Figure 3.

## 3. Discussion

We found that GABA_A_ receptors that incorporate the β3(N265M) mutation, co-expressed with α5 and γ2 subunits, are only partially insensitive to modulation by isoflurane. This result holds important implications for the mechanistic interpretation of behavioral experiments using animals that harbor this mutation.

### 3.1. Identification of Molecular Targets of Anesthetics

Despite many years of intense scientific study, elucidating the mechanisms by which general anesthetics produce their various effects remains an important unmet goal. The greatest progress has been made with injected anesthetics, which generally have higher potencies and act on a smaller range of molecular targets than the lower potency and more promiscuous inhaled agents. In large part, the difficulty for the inhaled agents is that their many molecular targets make it hard to know which matter.

This principle applies to injected drugs as well, including the “GABAergic” anesthetics propofol and etomidate; even these more specific drugs have been found to modulate additional molecular targets [20]. Thus, experiments showing that mutations of the GABA_A_R β2 or β3-subunit that attenuate or eliminate their modulation by anesthetics also confer resistance to anesthesia in behavioral studies provided important confirmation that GABA_A_Rs mediate the effects of these drugs [4,5]. In and of itself, this is an important conclusion. In addition, by mutating specific subunits and testing how each of them influences the various endpoints of anesthesia, it has been possible to link modulation of specific subtypes of GABA_A_Rs to specific endpoints of anesthesia: GABA_A_Rs that incorporate β2 subunits to sedation, and β3 to loss of righting reflex, respiratory depression, and immobilization [1,21]. These subunit-specific actions presumably correspond to differential expression of the β2 versus β3 subunits; though both are distributed throughout the brain, with a high degree of overlap in cortex and hippocampus, their expression levels do vary by brain region in some subcortical structures [22,23].

For the inhaled agents a similar strategy has been followed, but focusing primarily on α-subunits, because their mutation more consistently conferred resistance to inhaled anesthetics than β-subunit mutations [12,19]. Mutations of the α1 subunit modestly increased the concentration of isoflurane required to impair the righting reflex, but they did not alter its immobilizing or amnestic effects [7]. Mutations of the α2 subunit failed to alter the effects of isoflurane on the loss of righting reflex, immobility or amnesia [8]. Similarly, mice carrying β3-subunit mutations required slightly higher concentrations for immobility [5,9,10], but the loss of the righting reflex was unaltered. These limited effects on sensitivity to inhaled anesthetics stand in stark contrast to the findings for injected anesthetics; together they have been taken as an indication that modulation of GABA_A_Rs contributes little to the general anesthetic properties of inhaled agents.

### 3.2. Contribution of GABA_A_R Modulation to Memory Suppression

The report that mice carrying the β3(N265M) mutation are unaltered in their sensitivity to isoflurane [10] thus fits within this overall pattern of little, if any, contribution of GABA_A_Rs to inhaled anesthesia. However, it stands at odds with other results showing that β3-GABA_A_Rs are important for normal mnemonic function [24], and that modulation of β3-GABA_A_Rs by isoflurane can be strong—approximately one-third to one-half the modulatory effect of etomidate at equally amnestic concentrations [25]. Moreover, forebrain-specific conditional knockout of β3-GABA_A_Rs was found to confer strong resistance to isoflurane-induced amnesia in a hippocampus-dependent paradigm—fear conditioning to context [26]. In addition, β3-subunits pair primarily with α5-subunits, which are highly enriched in the hippocampus, are well-established contributors to memory, and have been shown to mediate amnesia produced by injected anesthetics. Therefore, these many circumstantial and direct indications that β3-GABA_A_Rs contribute to isoflurane-induced amnesia make it actually quite surprising that the β3(N265M) mice did not resist isoflurane in fear conditioning studies [10].

Our present findings provide a possible reconciliation of these results. Although the β3(N265M) mutation reduced the ability of isoflurane to potentiate GABA-elicited responses, potentiation still occurred: the increase in GABA-elicited responses for mutant receptors was approximately one-half that seen for wild-type receptors (Figure 1 and Figure 2). In the study by Liao et al. [10] the 50% effective concentration (EC50) of isoflurane needed to reduce freezing to context (a hippocampus-dependent task) was approximately 50% greater for mutant mice than for wild-type mice (0.19% wild-type vs. 0.28% mutant), though the difference did not reach statistical significance. Perhaps this is because the mutation did not fully prevent isoflurane modulation. Had the mutation been fully effective, how great an effect on memory would be expected? Supposing that isoflurane’s impairment of memory is entirely due to β3-GABA_A_R enhancement (which is unlikely), then the EC50 would have been increased by two-fold, or approximately twice that observed. Therefore, acknowledging that changes in sensitivity in vitro are not expected to translate quantitatively to changes in drug potency in vivo, these results indicate nevertheless that modulation of β3-GABA_A_Rs might contribute substantially to isoflurane’s ability to suppress memory.

A limitation of this study is that we tested the impact of the β3(N265M) mutation when co-expressed with only a single set of partner subunits (α5 and γ2L), and it is possible that the findings do not apply to all subunit combinations. Nevertheless, as α5 subunits are heavily expressed in hippocampus [15], they partner primarily with β3 subunits [27], and the great majority of synaptic as well as extrasynaptic receptors incorporate γ2 subunits [28], this subunit combination does constitute an important class of native receptors. A second limitation is that we conducted measurements of isoflurane modulation in vitro but not in vivo. The use of this approach facilitated the study of receptors with known composition under well-defined conditions, and it has been used widely in the past to characterize the influence of receptor mutation on pharmacological sensitivity [20]. However, it is possible that other factors might come into play in vivo, such as the transient concentration of neurotransmitter that activates receptors, or the presence of accessory subunits that are not present in HEK293 cells but that might modify isoflurane sensitivity. By the same token, there is no reason to expect that isoflurane sensitivity would differ.

The considerations outlined above do not, of course, prove that β3-GABA_A_Rs mediate isoflurane’s ability to impair memory, wholly or in part; or that they mediate any other endpoints of isoflurane anesthesia. They might, however, explain why several indirect and direct lines of evidence implicated β3-GABA_A_Rs in isoflurane-induced amnesia, an expectation that was not supported by findings from β3(N265M) mice. Future studies of these mice, which have proved so valuable in elucidating anesthetic mechanisms, should take this partial sensitivity to isoflurane into account.

## 4. Materials and Methods

### 4.1. Cell Culture and Receptor Expression

Cell culture materials were purchased from Invitrogen (Carlsbad, CA, USA) unless stated otherwise. Transformed human embryonic kidney 293 cells were purchased from American Type Culture Collection (Manassas, VA, USA) and cultured under standard conditions in minimum essential medium with L-glutamine supplemented with minimum essential medium amino acids solution (0.1 mM), sodium pyruvate (1 mM), penicillin-streptomycin and 10% fetal bovine serum in 60 × 15 mm dishes. After incubation in 5% CO_2_ at 37 °C for 24 h, the cells were transiently transfected with α5β3γ2L (1:1:5) or α5β3(N265M)γ2L (1:1:5) cDNA using OPTImem (Life Technologies, Carlsbad, CA, USA). GABA_A_R subunits were individually inserted in the mammalian expression vector pUNIV and enhanced green fluorescence protein (EGFP) using Lipofectamine 2000 (Invitrogen, Carlsbad, CA, USA) [29] After incubating for 24 h, the cells were re-plated with supplemented minimum essential medium onto 12-mm coverslips. Cells were ready for electrophysiological recordings 24–48 h post-transfection.

### 4.2. Electrophysiological Recordings

Coverslips with transfected cells were transferred to a culture dish filled with HEPES-buffered extracellular solution containing (mM): 145 NaCl, 5 KCl, 10HEPES, and 1.8 CaCl_2_, pH = 7.4. Dishes were placed on the stage of a Leica inverted microscope (Leica Microsystems, Buffalo Grove, IL, USA) with Hoffman-modulated optics. Glass pipettes were prepared from borosilicate glass (Warner Instruments, Hamden, CT, USA) with a multi-stage puller (Sutter Instruments, Novato, CA, USA) and fire polished. Recording pipettes were filled with (mM): 130 KCl, 10 HEPES, 5 EGTA, 1 MgCl_2_ and 5 MgATP. Open tip resistances were typically 2–5 MΩ for whole-cell recordings. Cells were visualized using a mercury arc lamp and an EGFP filter set. Data were acquired using equipment from Axon Instruments (now a division of Molecular Devices, Sunnyvale, CA, USA). Currents were recorded with the whole-cell configurations of the patch-clamp technique using an Axopatch 200A amplifier, sampled at 20 kHz (Digidata 1440A converter), filtered at 2 kHz (−3 dB, four-pole Bessel) and stored on a computer running pCLAMP 10.2 software (Molecular Devices, Sunnyvale, CA, USA). The chloride equilibrium potential was ~0 mV, resulting in inward currents at a holding potential of −40 mV.

### 4.3. Solution Preparation and Application

Solutions were prepared as described previously [30,31]. Briefly, GABA-containing solutions were prepared from a freshly made stock solution. Isoflurane-containing solutions were made using isoflurane-saturated air as a “stock”, added to the experimental solution in gas-tight Chemware© Teflon FEP gas sampling bags (North Safety Products, Cranston, RI, USA). The targeted concentrations of isoflurane were confirmed using a Varian 3700 gas chromatograph (Varian Inc., Walnut Creek, CA, USA) equipped with a flame ionization detector and an 80/100 Poropak Q packed stainless-steel column. Measured concentrations were all within ~5% of targeted concentrations.

Extracellular saline and test solutions containing GABA (Sigma, St. Louis, MO, USA) and/or isoflurane (Lancaster Synthesis Inc., Pelham, NH, USA) were transiently applied to fluorescing whole cells lifted from the coverslip and placed before flowing streams of extracellular solution. Solution exchange rates were estimated using the open tip junction potential method [32] to be ~20 ms. For solution exchanges between larger numbers of solutions that included the volatile compound isoflurane, we used a custom-fabricated multibarrel application device consisting of Teflon™ and polyimide tubing connected to closed gravity-fed glass syringes. We used a stepper motor-based microscope translation stage (Corvus, iTK, Dr. Kassen, GmbH, Lahnau, Germany). The solutions were delivered to single cells using a series of low volume, manually-controlled Teflon™ (DuPont, Wilmington, DE, USA) valves. Typically, multiple concentrations were tested in each cell.

Test solution pulses (500 ms) were applied at intervals that prevented accumulation of desensitization, manifested as a decline in peak current amplitudes triggered by ~EC10 concentrations of GABA (data not shown). Concentration–response relationships for α5β3γ2L and α5β3(N265M)γ2L receptors using these methods were published recently [18].

### 4.4. Data Analysis

Whole-cell currents were analyzed using Clampfit 10.4 (Molecular Devices, Sunnyvale, CA, USA), Origin 9.0 (OriginLab, Northampton, MA, USA) and Excel (Microsoft, Redmond, WA, USA). Results are presented as mean ± standard error of the mean. Numbers of observations reflect numbers of recordings obtained from different cells or patches. Students’ *t*-tests and z-tests were used to test for differences between group means as indicated in figure legends. Statistical significance was taken at *p* < 0.05. Multiple comparisons were corrected post-hoc with the Sidak–Holm test.
